# Quality Analysis of American Ginseng Cultivated in Heilongjiang Using UPLC-ESI^−^-MRM-MS with Chemometric Methods

**DOI:** 10.3390/molecules23092396

**Published:** 2018-09-19

**Authors:** Yong-Gang Xia, Yan Song, Jun Liang, Xin-Dong Guo, Bing-You Yang, Hai-Xue Kuang

**Affiliations:** Key Laboratory of Chinese Materia Medica, Heilongjiang University of Chinese Medicine, Ministry of Education, Harbin 150040, China; xiayonggang@hljucm.net or yonggangxia@163.com (Y.-G.X.); songyanharbin@163.com (Y.S.); lliangjunn@163.com (J.L.); guoxindong007@163.com (X.-D.G.); ybywater@163.com (B.-Y.Y.)

**Keywords:** *Panax quinquefolium*, ginsenosides, UPLC-MRM-MS, quantitative analysis, chemometric methods

## Abstract

American ginseng (*Panax quinquefolium*) has long been cultivated in China for the function food and medicine. Here, ultra-high performance liquid chromatography was coupled with electrospray ionization and triple quadrupole mass spectrometry (UPLC-ESI^−^-TQ-MS) for simultaneous detection of 22 ginsenosides in American ginseng cultivated in Mudanjiang district of Heilongjiang. The extraction conditions also were optimized by a Box Behnken design experiment. The optimized result was 31.8 mL/g as ratio of liquid to raw materials, 20.3 min of extraction time, and 235.0 W of extraction powers. The quantitative MS parameters for these 22 compounds were rapidly optimized by single factor experiments employing UPLC-ESI^−^-multiple reaction monitoring or multiple ion monitoring (MRM/MIM) scans. Furthermore, the established UPLC-ESI^−^-MRM-MS method showed good linear relationships (*R*^2^ > 0.99), repeatability (RSD < 3.86%), precision (RSD < 2.74%), and recovery (94–104%). This method determined 22 bioactive ginsenosides in different parts of the plant (main roots, hairy roots, rhizomes, leaves, and stems) and growth years (one year to four years) of *P. quinquefolium*. The highest total content of the 22 analytes was in the hairy roots (1.3 × 10^5^ µg/g) followed by rhizomes (7.1 × 10^4^ µg/g), main roots (6.5 × 10^4^ µg/g), leaves (4.2 × 10^4^ µg/g), and stems (2.4 × 10^4^ µg/g). Finally, chemometric methods, hierarchical clustering analysis (HCA) and partial least squares discrimination analysis (PLS-DA), were successfully used to classify and differentiate American ginseng attributed to different growth years. The proposed UPLC-ESI^−^-MRM-MS coupled with HCA and PLS-DA methods was elucidated to be a simple and reliable method for quality evaluation of American ginseng.

## 1. Introduction

American ginseng, the root of *Panax quinquefolium* L, belongs to the family Araliaceae and possesses the function of reducing blood sugar, regulating immunity, and decreasing tension [1,2,3,4]. It has been an important traditional Chinese medicine for more than 200 years (since the Qing Dynasty) [5]. American ginseng originated in southeast Canada and the northern USA, but it is now widely cultivated in most regions of China [6]. Many factors such as germplasm, soil, age, climate, and water source can greatly influence the growth of *P. quinquefolium* [7]. This may result in different chemical components in American ginseng and, thereby, diversity in its quality [8,9]. Heilongjiang province is located in the northeast of China, which has a unique natural climate and soil structure. The impact of this climate on American ginseng cultivated in Heilongjiang is worthy of further research. Therefore, it is necessary to identify and quantify these biological constituents to guarantee the quality, safety, and efficacy of American ginseng cultivated in the Heilongjiang province of China.

Ginsenosides are distinctive and cardinal ingredients that illuminate a series of pharmacological activities of American ginseng. These compounds can be used to assess the quality of its related products [10,11]. Many saponins have been isolated from American ginseng and its processed products, and most of them are naturally classified into protopanaxatriol (PPT), protopanaxadiol (PPD), oleanolic acid (OA), and ocotillol (OCO), according to their aglycones skeletons [12,13]. The contents of some saponins in *P. quinquefolium* may show a noticeable diversity with the following changes at cultivation ages and medical parts, and even natural climate and soil structure. In addition, it is reported that an ocotillol type of ginsenoside (pseudoginsenoside F11) is a chemical marker of American ginseng, to distinguish other *Panax* species, while ginsenoside Rf is a characteristic component of Asian ginseng [14,15,16]. High performance liquid chromatography (HPLC) methods [17,18] have been previously established for the detection of ginsenosides in different *Panax* species, but some methods suffer from low resolution, low sensitivity, and long analytical time. With superior performances in sensitivity and selectivity, ultra performance liquid chromatography coupled with tandem mass spectrometry (UPLC-MS/MS) has been confirmed to be a powerful tool for the analysis of active substances in traditional Chinese medicines [19,20,21,22]. Multiple reaction monitoring (MRM) in triple quadrupole mass spectrometry is frequently employed for quantitative analysis [15,23,24].

In this study, a new quality evaluation method based on UPLC-ESI^−^-MRM-MS is readily developed for the simultaneous determination of 22 ginsenosides in different medical parts (main roots, hairy roots, rhizomes, leaves, and stems) and growth years (one year to four years) of *P. quinquefolium*. It aims to determine the optimal harvesting time and the best medical parts of American ginseng cultivated in the Heilongjiang districts of China. The results may provide a reason for the sustainable development and utilization of this medicinal resource in order to serve the health of the people. It could also increase the economic income of local pharmaceutical farmers. Finally, it is demonstrated that the UPLC-ESI^−^-MRM-MS with chemometric methods is a credible tool to evaluate American ginseng cultivated in the Heilongjiang province of China.

## 2. Results and Discussion

### 2.1. Optimization of Separation and MS Conditions by UPLC-MIM/MRM-EPI

**Figure 1:**
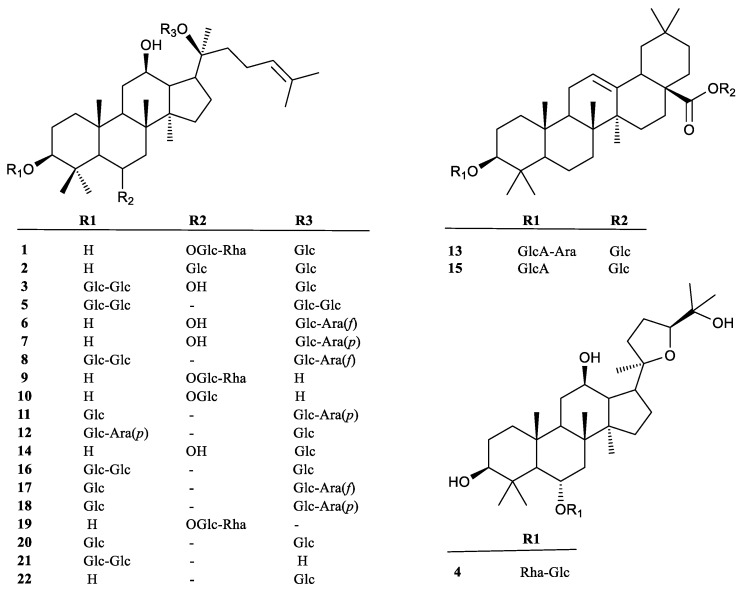
Chemical structures of the 22 investigated saponins in *P. quinquefolium* cultivated in the Heilongjiang province.

**Figure 2:**
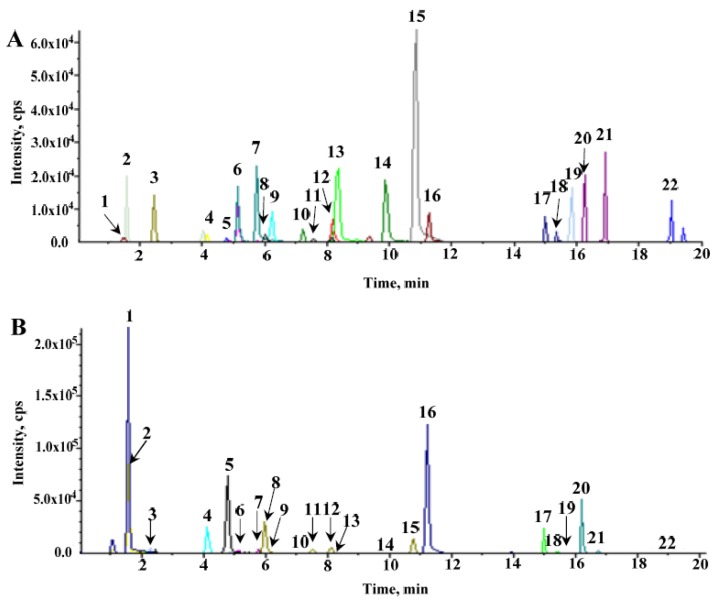
UPLC-MRM-MS TIC of reference standards (**A**) and the hairy root of American ginseng (**B**).

To obtain satisfactory chromatographic separations, UPLC conditions were optimized by selecting columns and adjusting the gradient elution for the separation of all of the compounds in this study. A Waters T3 column (1.8 µm, 2.1 × 150 mm) was selected for the analysis of 22 ginsenosides to achieve smooth baseline separation. Their structural information is shown in Figure 1. Formic acid was effective for chromatographic separation, but it was necessary to find a compromise for the ionization of the compounds under ESI^−^ modes. The results showed that 0.1% formic acid in both water and acetonitrile produce the highest MS intensity and the best resolutions for most of the peaks tested. Therefore, an optimized gradient elution program was applied for the separation of the 22 ginsenosides in 20 min, resulting from 0.1% formic acid in both water and acetonitrile at a flow rate of 0.3 mL/min with a column temperature of 30 °C. The total ion chromatography (TIC) of the 22 standard analytes is depicted in Figure 2A.

Fine tuning declustering potential (DP) and collision energy (CE) were conducted by the direct infusion of targeted substance into mass spectrometers. However, this method is time-consuming, labor-intensive, and a massive waste of standards [24,25]. In a previous report, a stepped MS^all^ technique determined the optimal CE values in triple time-of-flight MS, and then these optimal CE values were validated on a TQ-MS [24]. An issue was obviously seen in the simultaneous use of two different MS instruments.

However, in this study, all of the targeted compounds of the MS/MS parameters (precursors, product ions, DP, and CE) were rapidly optimized for each analyte by several multiple reaction monitoring (MRM) and multiple ion monitoring (MIM)-EPI runs in a UPLC-TQ-MS. The MRM and MIM scans were specialized and sensitive modes for the TQ-MS. Because DP values played an essential role in the formation of [M − H]^−^ or [M + HCOO]^−^ precursor ions, the DP values for each compound were investigated at 70, 80, 90, 100, 110, 120, and 130 V with a fixed CE value of 5 eV employing UPLC-MIM-IDA-EPI scans. The results are shown in Figure S1A, and the optimal DP values for the 22 ginsenosides were between 90 and 120 eV. Because the CE values could fragment [M − H]^−^ or [M + HCOO]^−^ precursor ions to give Q_3_ product ions, the different CE values (40, 60, 80, and 100 eV) were applied with fixed DP values to determine the optimal Q_1_/Q_3_ ion pairs and CE values using several UPLC-MRM-IDA-EPI scans. Here, the selection criterions of Q_3_ ions were characterized by both high intensity and good stability using appropriate CE values.

The [M–H − 162]^−^ ions were chosen as Q_3_ product ions for peaks **1**–**3**, **5**, **10**, **13**–**16**, and **20**–**22**, while the [M–H − 132]^−^ as Q_3_ ions were used for peaks **6**–**8**, **11**, **12**, **17**, and **18**. With regard to peaks **4**, **9**, and **19**, the [M–H − 146]^−^ ions were readily suitable for quantitative Q_3_ ions. The optimal CE values were further investigated using Q_1_/Q_3_ ion pairs in MRM mode, based on estimating their values as medians with an interval of 5 V. As shown in Figure S1B,C, the CE values of compounds **2**, **4**, **6**, **7**, **9**, **10**, **14**, and **17**–**22** were determined to be between 40 and 55 eV, but there were relatively large molecular weights of ginsenosides **1**, **3**, **5**, **8**, **11**–**13**, and **16**, and the ranges of the CE values were applied between 60 to 75 eV. The results of all optimal parameters for the 22 ginsenosides are shown in Table 1. This method showed that it could reconcile the optimal instrumental parameters obtained by direct infusions being different from those in real sample analysis.

### 2.2. Optimization of Saponin Extraction

In this study, we used the Box-beckon design (BBD), the most popular form of response surface methodology (RSM), to optimize the extraction conditions and to obtain the highest content of ginsenosides. Thus, the total peak areas of the 10 most intensive compounds were selected as an index to evaluate the total extraction yields of ginsenosides.

The effects, such as extraction temperatures, extraction time, ultrasonic power, ratio of liquid to raw materials, and ethanol concentrations were systematically investigated by the single factor method. Figure S2 showed that ultrasonic temperatures (70–90 °C) and ethanol concentrations (80–100%) had weak effects on extraction yields of these active compounds. Thus, extraction time, ultrasonic power, and the ratio of liquid to raw materials were selected as the BBD factors. On the basis of single-factor experimental results, 15 min to 25 min for extraction time, 200 W to 300 W for ultrasonic power, and 20 mL/g to 40 mL/g for the ratio of liquid to raw materials were used in a BBD method to design the experimental project.

The BBD experimental results showed that a second-order polynomial equation was fitted to analyze the data. A mathematical regression model about Y was obtained, as follows:

Y = 3.869 × 10^7^ + 2.957 × 10^6^ × X_1_ + 6.761 ×10^4^ × X_2_ − 3.909 × 10^5^ × X_3_ − 4.240 × 10^5^ × X_1_X_2_ − 1.365 × 10^6^ × X_1_X_3_ − 2.487 × 10^5^ × X_2_X_3_ − 9.085 × 10^6^ × X_1_^2^ − 5.219 × 10^5^ × X_2_^2^ − 1.124 × 10^6^ × X_3_^2^.

Here, Y is the response, which was the overall peak intensity of 10 target compounds, and X_1_, X_2_ and X_3_ were assigned to test the variables of the ratio of liquid to raw materials, extraction time, and ultrasonic power, respectively.

The three-dimensional profiles of the multiple non-linear regression models were depicted in Figure S3. The results displayed that that ratio of liquid to raw materials played a more prominent effect on the extraction yields than those of the extraction time and ultrasonic power. By applying the second-order polynomial equation, prediction optimization values were evaluated, and the result was that X_1_ (ratio of liquid to raw materials) was 31.8 mL/g, X_2_ (extraction time) was 20.3 min, and X_3_ (ultrasonic powers) was 235.0 W. Finally, to validate the optimal conditions prepared via RSM, parallel experiments were performed three times to verify the predicted optimum conditions. The predicted values were in excellent agreement with the experimental results.

### 2.3. Qualitative Analysis of Investigated Ginsenosides in Cultivated P. quinquefolium

As shown in the total ion chromatography of hairy roots (Figure 2B), comparison of their retention time with those of the authentic saponin standards, peaks **1**–**22** were unambiguously identified as ginsenoside Re, Rg1, vinaR4, pseudo F_11_, Rb_1_, F5, F3, Rc, 20-R-Rg2, 20-R-Rh1, Rb2, Rb3, pseudo-Rt1, F1, chikusetsu saponin Iva, Rd, notoginseng-Fe, Rd2, F4, F2, Rg3, and CK, respectively. Their major fragment ions are described in Figure S4 and Table S1, respectively. The ESI^−^-MS/MS spectra were obtained from fragmentation of [M − H]^−^ or [M + HCOO]^−^ precursors, and the results exhibited a successive loss of the glycosidic units until the formation of [aglycone − H]^−^ ions. According to the structural properties, the PPT type compounds had an aglycone ion at *m*/*z* 475, which was evident in peaks **1**–**3**, **6**, **7**, **9**, **10**, **14**, and **19**. The MS/MS spectrum, for Rg1 (Figure S4A; peak **2**) gave [PPT − H]^−^ at *m*/*z* 475.4 via the successive elimination of two glucosyl groups. However, PPD-type saponins, peaks **5**, **8**, **11**, **12**, **16**–**18**, and **20**–**22**, produced an aglycone ion at *m*/*z* 459. The MS/MS spectrum of Rd2 (Figure S4B, peak **18**) produced a [PPD − H]^−^ at *m*/*z* 459.4 via continuous losses of an arabinosyl (132 Da), a glucosyl (162 Da), and a glucosyl (162 Da). Meanwhile, the OA-type compounds, including peaks **13** and **15**, produced a *m*/*z* 455 [OA − H]^−^ aglycone ion. For instance, chikusetsu saponin Iva (peak **15**) shown in Figure S4C, gave diagnostic aglycone at *m*/*z* 455.4, which was formed through the losses of a glucosyl (162 Da) and a GlcUA (176 Da). In contrast, compound **4** (Figure S4D) was OCO type, and it yielded an aglycone ion at *m*/*z* 491.3, which was formed by the losses of a rhamnosyl (146 Da) and a glucosyl (162 Da). Therefore, these diagnostic product ions could be used for the identification of triterpenoid aglycones. The corresponding neutral loss could be further employed to determine sugar unit moieties.

### 2.4. Quantitative Analysis of Real Samples

#### 2.4.1. Validation of Analytical Methods

As shown Table 2, all coefficients of determination (*R*^2^) higher than 0.995 for all the ginsenosides **1**–**22** displayed good linearity over certain concentration ranges. The limits of quantification (LOQ) and the limits of detection (LOD) for all of the investigated compounds are shown in Table 2. The RSDs of inter- and intra-day precisions (*n* = 6) were less than 2.72% and 2.74%, respectively. This showed good precision for the entire method. The compounds showed to be good stability with a variation of 0.19–3.86% at least 6 h at room temperature. In addition, the sample recoveries ranged from 97–105% for all sample with an RSD within 4% (Table S2). A matrix effect was assayed by relative recoveries in methanol solvent for all compounds ranged between 96% and 103%, thus showing the minimal matrix suppression or enhancement. Hence, this verified UPLC-ESI^−^-MRM-MS method was feasible for the quantitative assessment of *P. quinquefolium*.

#### 2.4.2. Distribution of Compounds **1**–**22** in Different Parts from *P. quinquefolium*

**Figure 3:**
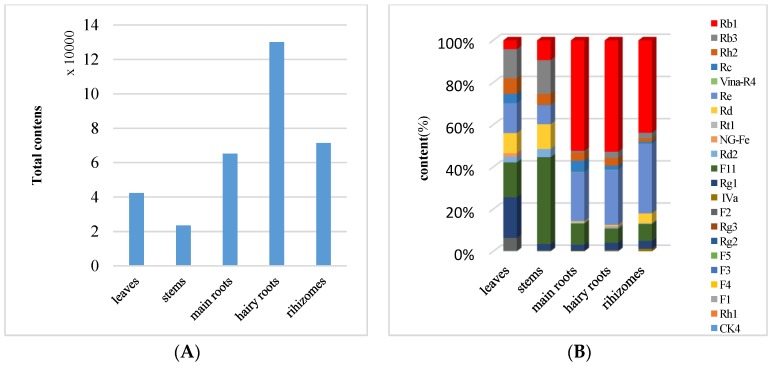
(**A**) Total content of 22 investigated ginsenosides detected in different plant parts of *P. quinquefolium*; (**B**) Graphic representation of 22 investigated ginsenosides in different plant parts of *P. quinquefolium*.

In this study, 22 bioactive ginsenosides were quantified in main roots, hairy roots, rhizomes, leaves, and stems of *P. quinquefolium* cultivated in Heilongjiang province by employing a developed UPLC-ESI^−^-MRM-MS method. Therefore, we studied these 22 compounds in different plant parts, which could be calculated using the corresponding calibration curve and data, as listed in Table S3. A graphical representation of this observation is shown in Figure 3A,B, which indicated their remarkable content variations. The highest total content of the 22 analytes was in the hairy roots (1.3 × 10^5^ µg/g), followed by the rhizomes (7.1 × 10^4^ µg/g), the main roots (6.5 × 10^4^ µg/g), leaves (4.2 × 10^4^ µg/g), and stems (2.4 × 10^4^ µg/g) (Figure 3A). These results indicated that the contents of the fibrous roots were five-fold that of the stems, which agreed with previous reports [26]. However, compared to *P. notoginsen* in literature, the higher content of the total ginsenosides was located in the rhizome, followed by the main roots and hairy roots [27]. As a contrast, the content of total ginsenosides was higher in the leaves and hairy roots than in other parts of *P.* *ginseng* [28].

As shown in Figure 3B and Table S3, it was further observed that ginsenoside Re (**1**), Rg1 (**2**), pseudo-F11 (**4**), Rb1 (**5**), Rc (**8**), Rb2 (**11**), and Rb3 (**12**), were major constituents in the roots, especially in the hairy roots. However, some trace compounds **6**, **7**, **9**, **10**, **13**, **14**, **19**, and **22** were also detected in this study. In previous studies, pseudo-F11 (**4**) was a typical marker constituent of American ginseng [15,29]. Pseudo-F11 (**4**) was detected in all parts of cultivated *P. quinquefolium* and the content of peak **4** (9677 µg/g) in stems was much higher than in the hairy roots (8744.3 µg/g), leaves (6992.2 µg/g), roots (6528.5 µg/g), and rhizomes (5737.6 µg/g). In contrast, the contents of peaks **2** (8186 µg/g), **12** (5841 µg/g), **16** (4046 µg/g), **18** (1122 µg/g), and **20** (2421 µg/g) in the cultivated plant leaves were relatively higher than those in main roots, hairy roots, and rhizomes. Nevertheless, the content of peak **5** (1773 µg/g) in the leaves was relatively low.

### 2.5. Multivariate Statistical Analysis of P. quinquefolium in Different Growth Years

#### 2.5.1. Hierarchical Clustering Analysis

**Figure 4:**
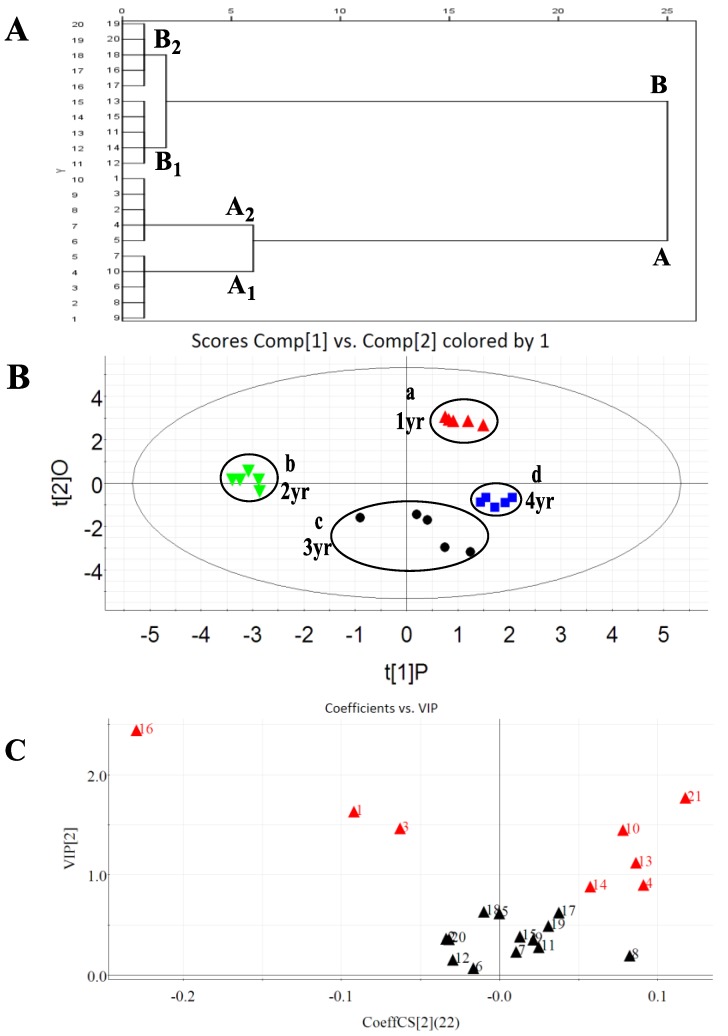
(**A**) HCA of different growth years of *P quinquefolium*; (**B**) PLS-DA score of different growth years of *P. quinquefolium*; (**C**) VIP plot of the 22 ginsenosides based on PLS-DA.

Hierarchical clustering analysis (HCA) emphasizes relatively homogeneous clusters of samples based on measured characteristics for the present data. HCA was performed on the data from samples grown in different years. By employing Ward’s method with the Euclidean distance as measurement indicators, the samples were categorized based on the content of 22 investigated saponins. The dendrogram of HCA is shown in Figure 4A, from which the quality characteristics could be revealed more clearly. The 20 samples were grouped into two main clusters. Namely, samples 1–10 were in cluster **A** from *P. quinquefolium* grown in Year 1 to Year 2, and the others were in cluster **B** from Year 3 to Year 4; both were further clustered into two corresponding subgroups, respectively. In this case, samples 1–5, 6–10, 11–15, and 16–20 were clearly labelled as **A**_1_, **A**_2_, **B**_1_, and **B**_2_, respectively. From Figure 4A, it could be easily seen that all the tested samples that were collected from the same years were unambiguously classified into a cluster, which indicated that the quality of these samples were homogenous and stable.

#### 2.5.2. Partial Least Squares Discrimination Analysis

The content distribution of the 22 investigated ginsenosides in roots as a function of different growth years was visualized by performing a partial least squares discrimination analysis (PLS-DA) in Figure 4B,C. PLS-DA is a supervised pattern recognition method using chemometrics to compress the data and to extract information. PLS-DA was employed to compare and evaluate the quality of *P. quinquefolium* roots grown for one to four years based on the content of 22 targeted ginsenosides. The PLS-DA score plot of the final combined dataset is illustrated in Figure 4B, and it was obvious that these 20 samples were distinctively grouped into four clusters a–d on the basis of their different years as assigned to *P. quinquefolium* roots grown in 1, 2, 3, or 4 years, respectively. This means the holistic qualities of *P. quinquefolium* roots grown in the same number of years were consistent with each other. The different growth years indeed produced diverse levels and variations of the investigated ginsenosides.

The variable importance plot (VIP) (Figure 4C) was used to identify the most relevant variables that were distinguished among the one year and four year samples. The VIP values presented the significance of variables in the PLS-DA model and with respect to Y. Here, a relatively larger VIP value was defined at 0.8, so a total of eight potential makers were readily discovered as ginseng Re (**1**), vina R4 (**3**), pseudo-F11 (**4**), Rh1 (**10**), pseudo-Rt1 (**13**), F1 (**14**), Rd (**16**), and Rg3 (**21**). Meanwhile, these markers should play essential roles in the discrimination of *P. quinquefolium* roots in different growth years. Table S3 shows that the total contents of these marker saponins for a 3 year sample reached the maximum value, which were 2-, 1.5-, and 1-folds that of 1 year, 2 year, and 4 year samples, respectively. Furthermore, the total contents of ginsenosides **1** and **16** showed the maximum value in the 3 year samples, whereas the content of pseudo-F11 (**4**) in 2 year was higher than that in other ages. These obvious variations could result from the specific climates and soil characteristics of the Heilongjiang province of China.

## 3. Experimental

### 3.1. Plants and Reagents

*P. quinquefolium* plants were collected in September 2016, from Mudanjiang (43°49′–44°35′ N, 129°46′–130°32′ E), Heilongjiang, China. All parts of *P. quinquefolium* were dried naturally in the shade and achieved a constant weight. Ginsenosides Re, Rg1, vina R4, pseudo F11, Rb1, F5, F3, Rc, Rg2, Rh1, Rb2, Rb3, pseudo-Rt1, F1, chikusetsu saponin Iva, Rd, notoginseng (NG)-Fe, Rd2, F4, F2, Rg3, and CK were purchased from Chengdu Must Bio-technology Co. (Chengdu, China). The structural information of these compounds were observed in Figure 1.

HPLC-grade formic acid and acetonitrile were supplied from Fisher Scientific (Watham, MA, USA). Deionized water was obtained by a Mill-Q system, purchased from Millipore, Billerica, MA, USA. Other chemicals were of analytical purity.

### 3.2. Extraction Procedures

Ultrasound-assisted extraction was carried out in an ultrasonic device with a thermostat. *P. quinquefolium* (0.1 g) was extracted with 3.2 mL of 80% ethanol, 70.0 °C and 235.0 W for 20.3 min. Then, the samples were centrifuged at 12,000 rpm for 10 min to remove the insoluble materials. The supernatants were filtered through a 0.22 µm nylon syringe filter before being injected into the UPLC system for analysis.

### 3.3. UPLC-MS/MS System

An Acquity UPLC H-Class system (Waters Corp., Milford, MA, USA) was applied for analysis. A HSS T3 column (2.1 × 150 mm, 1.8 μm) equipped with a HSS T3 guard column (2.1 × 5 mm, 1.8 μm) was employed to achieve separation at 35 °C. The binary system phase consisted of solvent A (water with 0.1% formic acid) and solvent B (acetonitrile with 1.0% formic acid). The gradient elution was used as follows: 0–6 min, 30–35% B; 6–12 min, 35–40% B; 12–16 min, 40–75% B; 16–21 min, 75–85% B, 21–26 min, 85–95% B, 26–26.1 min, 95–30% B. Other parameters were: flow rate 0.3 mL/min, injection volume 2.0 μL, and sample room temperature 10 °C.

A 4000 Qtrap mass spectrometer (SCIEX Corp., Framingham, MA, USA) with an electrospray ionization (ESI) interface was utilized for Mass spectrometric analysis in negative ion mode. All instrument data were acquired and processed by Analyst software (version 1.6, SCIEX). The parameters were set for all analytes in negative mode as follows: an ion spray voltage of 5500 V, a turbo spray temperature of 300 °C, and the interface heater was on. The pressures of the heater gas and the nebulizer gas were set to 40 psi. Nitrogen was used as a nebulizer and an auxiliary gas. Other parameters were set, such as an entrance potential (EP) and a cell exit potential (CXP) of 15 and 10, respectively.

### 3.4. Validation of Methods

A mixed stock solution containing all 22 compounds was obtained in methanol. A series of appropriate concentrations of working solutions were prepared by dilutions with methanol. The LOQ and LOD of each compound under same chromatographic conditions were determined at an S/N (Signal/Noise) of about 10 and 3, respectively.

The measurements of interday and intraday precisions were employed to determine the precision by analyzing the sample six times within a single day, and over three consecutive days for all mixed standards, respectively. In order to test its repeatability, six sample solutions (*P. quinquefolium* hairy roots) were prepared under the same condition, and then they were extracted by ultrasound assisted methods, and determined by UPLC-MS/MS, as mentioned above. A recovery was used to assess the accuracy of the analysis method by employing the standards addition method. Known amounts of reference standards (approximately equivalent to 80%, 100%, and 120% levels of each compound) were added into the samples with the same procedures, including extraction, and analyzed by UPLC-MS/MS. Each set of addition was repeated three times. The extraction recovery was calculated as follows: recovery = (sample contents after adding − original contents)/contents of standard solutions for adding.

### 3.5. Data Processing

Chemometric methods were used to evaluate the cultivated American ginseng in different growth years. The HCA was carried out using SPSS version 16.0 (SPSS, Inc., Chicago, IL, USA). Meanwhile, PLS-DA was performed by employing Masslynx 4.1 extended statistics (Waters MS Technologies, Milford, MA, USA).

## 4. Conclusions

A simple and feasible UPLC-ESI^−^-MRM-MS method was for the first time developed and validated for the simultaneous quantification of 22 saponins in different medicine parts and growth years of *P. quinquefolium* cultivated in the Heilongjiang province in northeast China. By comparison with other previous analytical methods, the major merits of this investigation can be further summarized as followed: (1) simultaneous determination of a large and trace amount of chemical compositions up to 22 ginsenosides containing PPT, PPD, OA, and OCO types, along with multiple pairs of structural isomers; (2) rapid determination of the optimal DP and CE values of 22 compounds by single-factor experiments employing UPLC-ESI^−^-MRM/MIM scans; (3) direct application of optimal DP and CE parameters in real sample analysis due to injection solutions during the process of online optimization with the same as those used in the sample. The UPLC-ESI^−^-MRM-MS results showed that the total contents of all 22 compounds were the most abundant in hairy roots followed by rhizomes, main roots, leaves, and stems. Comparative analysis of these major bioactive compounds in different medical parts indicated that it was quite significant for in-depth exploitation of leaves and stems from American ginseng cultivated in the Heilongjiang districts of China. Thus, this valuable medical plant resource will be sustainably utilized and will serve the health of the people. It could also increase the economic income of local pharmaceutical farmers.

## Figures and Tables

**Table 1:** The optimized MRM parameters and transitions for each analyst in UPLC-ESI-MRM-MS.

Peaks ^1^	Adducts	Parents (*m*/*z*)	Daughters (*m*/*z*) (Q/I) ^2^	DP (V)	CE (eV)
**1**	[M + HCOO]^−^	991.5	783.2/621.2	−110	−60
**2**	[M + HCOO]^−^	845.5	637.2/475.2	−120	−50
**3**	[M + HCOO]^−^	1007.5	799.3/475.3	−100	−60
**4**	[M + HCOO]^−^	799.2	653.0/491.2	−100	−50
**5**	[M + HCOO]^−^	1153.6	945.4/783.2	−100	−75
**6**	[M + HCOO]^−^	815.5	637.2/475.2	−120	−40
**7**	[M + HCOO]^−^	815.5	637.2/475.2	−120	−40
**8**	[M + HCOO]^−^	1123.6	945.4/783.4	−100	−65
**9**	[M + HCOO]^−^	829.5	637.2/475.2	−90	−45
**10**	[M + HCOO]^−^	683.4	475.2/637.2	−100	−40
**11**	[M + HCOO]^−^	1123.6	945.4/783.4	−110	−60
**12**	[M + HCOO]^−^	1123.6	945.4/783.4	−100	−65
**13**	[M − H]^−^	925.5	763.2/613.1	−120	−65
**14**	[M + HCOO]^−^	683.4	475.2/637.1	−100	−40
**15**	[M − H]^−^	793.4	631.1/455.2	−120	−65
**16**	[M + HCOO]^−^	991.5	783.2/621.2	−100	−65
**17**	[M + HCOO]^−^	961.5	783.2/621.3	−120	−45
**18**	[M + HCOO]^−^	961.5	783.2/621.2	−120	−45
**19**	[M + HCOO]^−^	811.5	619.4/457.1	−100	−45
**20**	[M + HCOO]^−^	829.5	621.4/459.2	−100	−40
**21**	[M + HCOO]^−^	829.5	621.4/459.2	−100	−55
**22**	[M + HCOO]^−^	667.4	459.4/621.4	−100	−40

^1^: **1**. G-Re; **2**. G-Rg1; **3**. Vina-G-R4; **4**. Pseudo-G-F11; **5**. G-Rb1; **6**. G-F5; **7**. G-F3; **8**. G-Rc; **9**. G-Rg2; **10**. G-Rh1; **11**. G-Rb2; **12**. G-Rb3; **13**. Pseudo-G-Rt1; **14**. G-F1; **15**. ChikusetsuIva; **16**. G-Rd; **17**. NG-Fe; **18**. G-Rd2; **19**. G-F4; **20**. G-F2; **21**. G-Rg3; **22**. G-CK. G: Ginsenoside; NG: notoginsenoside. ^2^: Q: transitions for quantification; I: transitions for identification.

**Table 2:** Calibration curves and detection limit of 22 saponins in *P. quinquefolium*.

Peaks	*t*_R_^1^	Regression Equations	*R* ^2^	Linear Ranges ^2^	LOD ^2^	LOQ ^2^
**1**	1.47	*y* = 10,699*x* + 337	0.9992	31–1000	1.95	3.91
**2**	1.57	*y* = 76,280*x* + 864	0.9976	34–1090	2.13	4.26
**3**	2.42	*y* = 86,118*x* − 716	0.9952	16–525	7.81	15.63
**4**	4.03	*y* = 17,787*x* + 569	0.9967	31–1000	1.91	3.90
**5**	4.79	*y* = 6627.9*x* + 25	0.9968	63–2000	7.81	15.63
**6**	5.11	*y* = 86,679*x* − 1514	0.9991	15–475	2.01	4.02
**7**	5.72	*y* = 184,024*x* − 1321	0.9950	8–515	1.01	2.01
**8**	5.98	*y* = 26,538*x* + 138	0.9982	14–450	1.76	3.52
**9**	6.20	*y* = 1,000,000*x* − 316	0.9997	4–225	1.95	3.91
**10**	7.19	*y* = 39,302*x* − 79	0.9950	34–550	4.29	8.59
**11**	7.56	*y* = 4487.7*x* + 138	0.9970	138–2200	34.78	68.75
**12**	8.10	*y* = 7394.2*x* − 221	0.9953	61–1960	15.31	30.63
**13**	8.29	*y* = 241,665*x* − 183	0.9996	9–275	1.95	3.91
**14**	9.86	*y* = 171,530*x* + 43	0.9965	8–250	0.98	1.95
**15**	10.80	*y* = 539,951*x* + 1515	0.9997	9–275	0.52	1.07
**16**	11.22	*y* = 80,694*x* + 567	0.9976	31–1000	1.95	3.91
**17**	14.99	*y* = 52,124*x* + 336	0.9996	16–500	1.95	3.91
**18**	15.34	*y* = 6715.3*x* + 552	0.9979	63–2020	15.78	31.56
**19**	15.82	*y* = 88,679*x* + 137	0.9980	15–475	1.86	3.71
**20**	16.26	*y* = 152,918*x* − 781	0.9994	16–500	1.93	3.96
**21**	16.91	*y* = 107,317*x* + 1172	0.9966	7–248	0.97	1.93
**22**	19.02	*y* = 60,120*x* + 92	0.9989	15–475	3.71	7.42

^1^, min; ^2^, ng/mL.

## Supplementary Materials

The following are available online, Figure S1: Single factor optimizations of DP (**A**) and CE (**B** and **C**) by UPLC-MIM/MRM-EPI. Figure S2: Effect of temperature (**A**), ethanol concentration (**B**), ultrasonic time (**C**), ultrasonic power (**D**), and ratio of liquid to raw materials (**E**) on extraction efficiency. Figure S3: Response surface plots showing the predicted value of total saponins yield. Figure S4: MS/MS spectra and fragmentations of peaks for **2** (**A**), **18** (**B**), **15** (**C**) and **4** (**D**). Table S1: Chemical information and major fragment ions of 22 ginsenosides. Table S2: Precision, stability, and recovery of 22 saponins in *P. quinquefolium*. Table S3: Contents of 22 compounds from different medicine parts and growth years in *P. quinquefolium* cultivated in Heilongjiang province.
