# Supplementary material for: Quality Analysis of American Ginseng Cultivated in Heilongjiang Using UPLC-ESI−-MRM-MS with Chemometric Methods
Source: Molecules. 2018 Sep 19;23(9):2396. doi: 10.3390/molecules23092396 (PMC6225424; doi:10.3390/molecules23092396)
Supplement: Supplementary file 1 [file molecules-23-02396-s001.pdf]

A

B

C

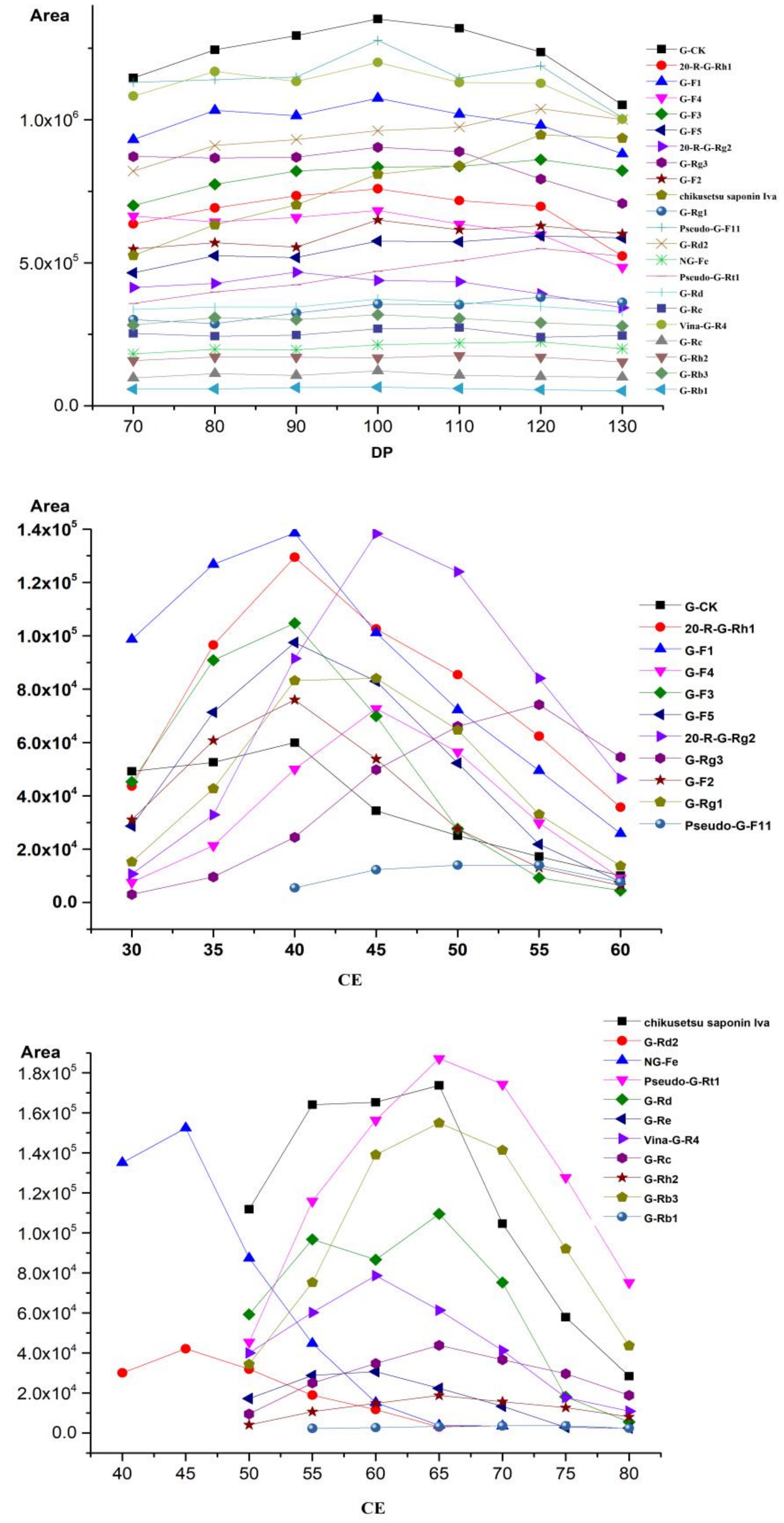


**Figure S1**. Single factor optimizations of DP by UPLC-MIM-EPI (**A**); Optimizations of CE by UPLC-MRM-EPI (**B** and **C**).

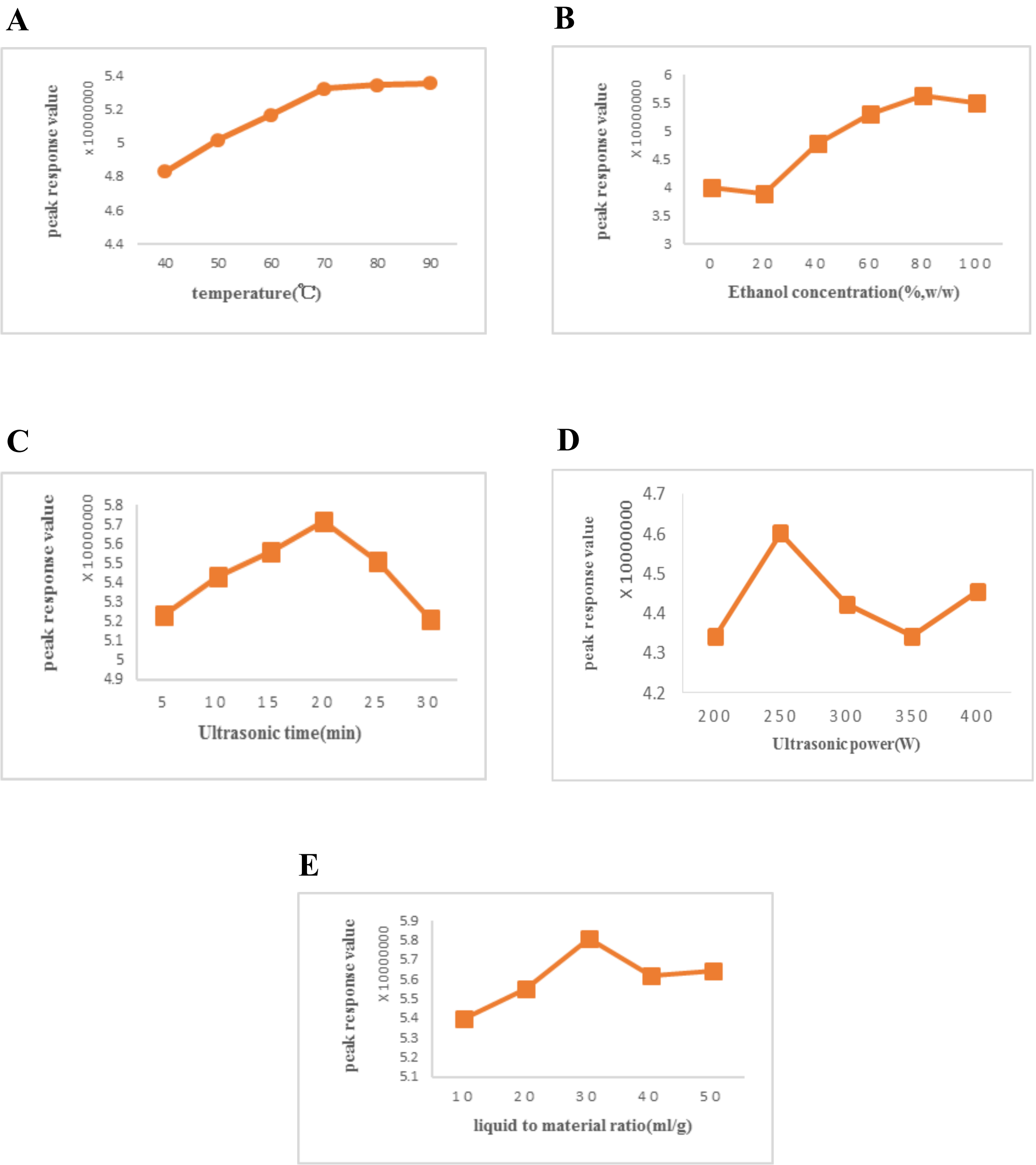


**Figure S2**. Effect of (**A**) temperature (℃), (**B**) Ethanol concentration (%, v/v), (**C**) Ultrasonic time (min), (**D**) Ultrasonic power (W), (**E**) Ratio of liquid to raw materials (mL/g) on extraction efficiency of the investigated compounds.

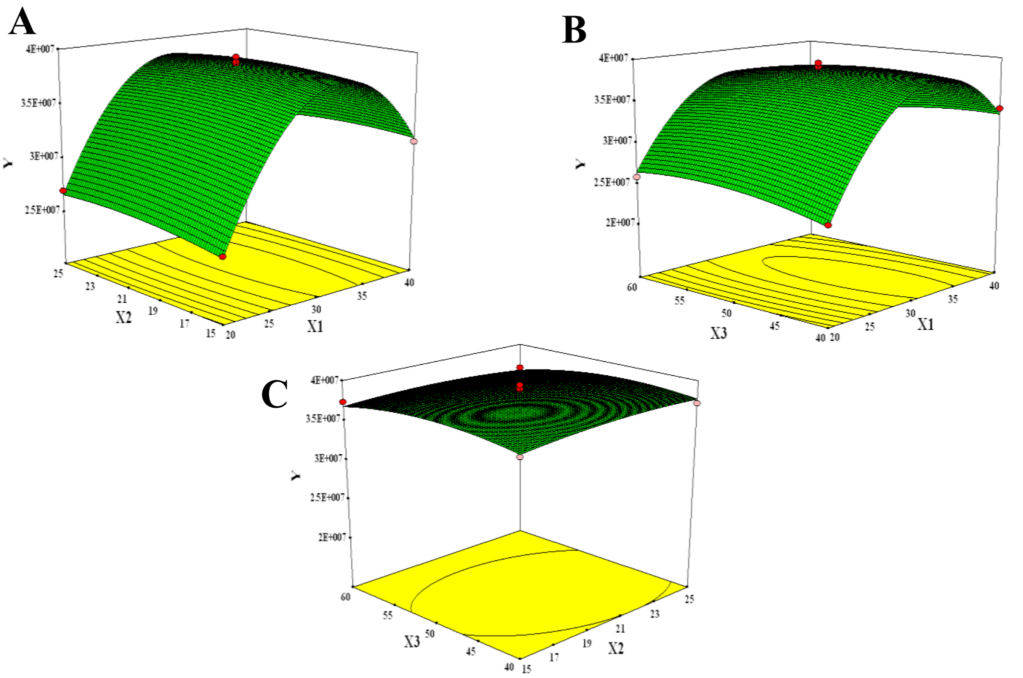


**Figure S3**. Response surface plots showing the predicted value of overall saponins yield. (A) Extraction time ($X_2$) vs ratio of liquid to raw materials ($X_1$), ultrasonic powers ($X_3$) is held at its optimum. (B) Liquid to raw materials ($X_1$) vs ultrasonic powers ($X_3$), extraction time ($X_2$) is held at its optimum. (C) Extraction time ($X_2$) vs ultrasonic powers ($X_3$), liquid to raw materials ($X_1$) is held at its optimum.

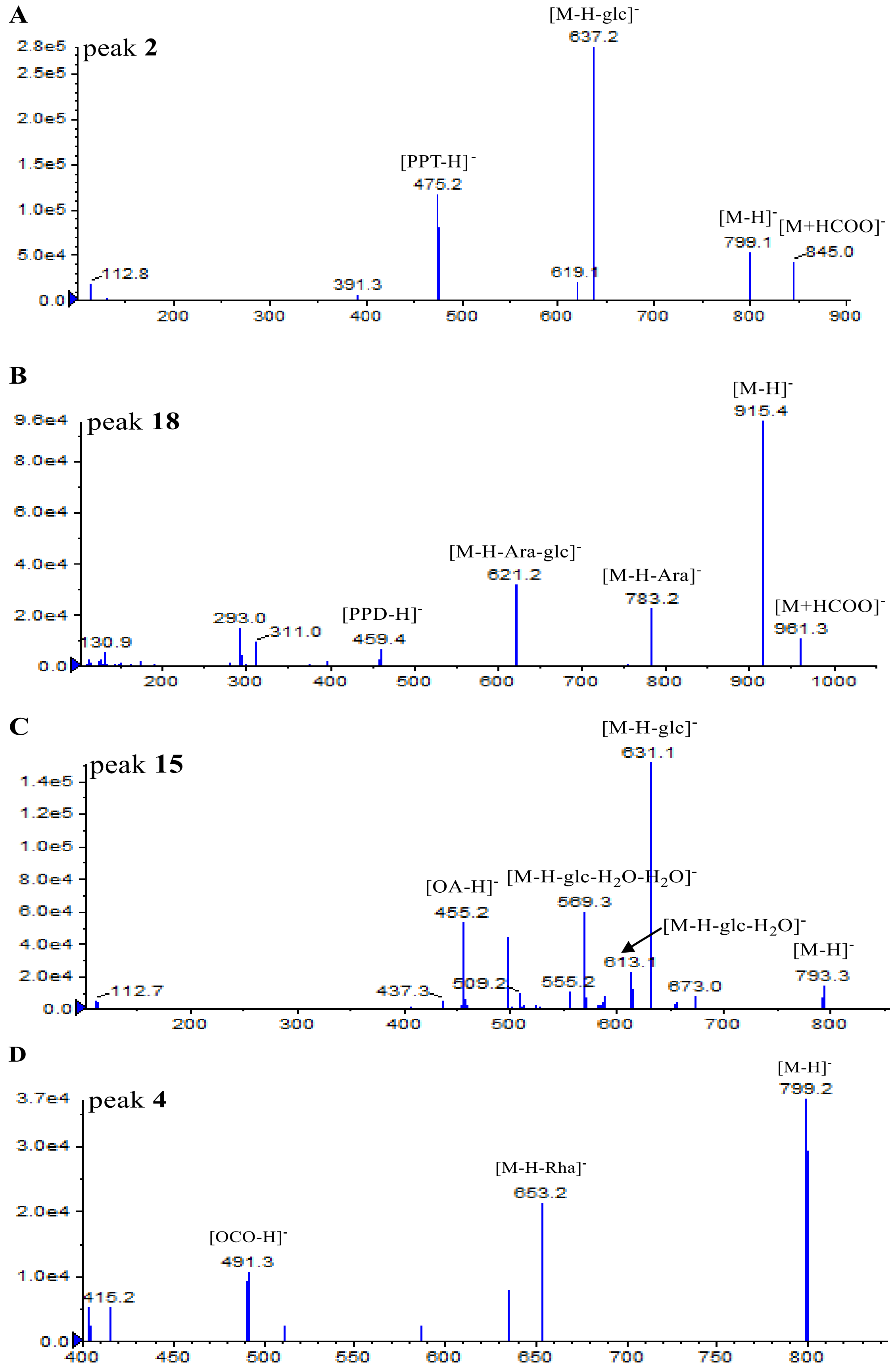


**Figure S4**. MS/MS spectra and fragmentations of peaks for **2** (**A**), **18** (**B**), **15** (**C**) and **4** (**D**).

**Table S1.** Chemical information and major fragment ions of 22 ginsenosides in *P. quinquefolium*.

| Peak | $t_R$ (min) | Identification | Measured mass (*m*/*z*) | Molecular formula [M-H]$^-$ | MS$^2$ fragment ions |
|---|---|---|---|---|---|
| 1 | 1.47 | Re | 991.5 | $C_{48}H_{81}O_{18}$ | 945.2 [M-H]$^-$, 799.0 [M-H-Rha]$^-$, 637.1 [M-H-Rha-Glc]$^-$, 619.0 [M-H-Rha-Glc-$H_2O$]$^-$, 475.1 [PPT aglycone-H]$^-$ |
| 2 | 1.57 | Rg1 | 845.5 | $C_{42}H_{71}O_{14}$ | 799.1 [M-H]$^-$, 637.2 [M-H-Glc]$^-$, 619.1 [M-H-Glc-$H_2O$]$^-$, 475.2 [PPT aglycone-H]$^-$ |
| 3 | 2.42 | Vina-R4 | 1007.5 | $C_{48}H_{81}O_{19}$ | 961.3 [M-H]$^-$, 799.3 [M-H-Glc]$^-$, 781.3 [M-H-Glc-$H_2O$]$^-$, 637.3 [M-H-2Glc]$^-$, 619.3 [M-H-2Glc-$H_2O$]$^-$, 475.3 [PPT aglycone-H]$^-$ |
| 4 | 4.03 | F11 | 799.2 | $C_{42}H_{71}O_{14}$ | 653.2[M-H-Rha]$^-$, 491.3 [OCO aglycone-H]$^-$ |
| 5 | 4.73 | Rb1 | 1153.6 | $C_{54}H_{91}O_{23}$ | 945.4 [M-H-Glc]$^-$, 783.5 [M-H-2Glc]$^-$, 621.4 [M-H-3Glc]$^-$, 459.4 [PPD aglycone-H]$^-$ |
| 6 | 5.11 | F5 | 815.5 | $C_{41}H_{69}O_{13}$ | 769.2 [M-H]$^-$, 637.2 [M-H-Ara]$^-$, 475.2 [PPT aglycone-H]$^-$ |
| 7 | 5.72 | F3 | 815.5 | $C_{41}H_{69}O_{13}$ | 769.2 [M-H]$^-$, 637.3 [M-H-Ara]$^-$, 619.2 [M-H-Ara-$H_2O$]$^-$, 475.3 [PPT aglycone-H]$^-$ |
| 8 | 5.98 | Rc | 1123.6 | $C_{53}H_{89}O_{22}$ | 1077.0 [M-H]$^-$,945.2 [M-H-Ara(p)$^{5)}$]$^-$, 783.2 [M-H-Ara(p)-Glc]$^-$, 621.2 [M-H-Ara(p)-2Glc]$^-$, 459.0 [PPD aglycone-H]$^-$ |
| 9 | 6.20 | Rg2 | 829.5 | $C_{42}H_{71}O_{13}$ | 783.1 [M-H]$^-$, 637.2 [M-H-Rha]$^-$, 475.2 [PPT aglycone-H]$^-$ |
| 10 | 7.19 | Rh1 | 683.4 | $C_{36}H_{61}O_9$ | 637.3 [M-H]$^-$, 475.3 [PPT aglycone-H]$^-$, |
| 11 | 7.56 | Rb2 | 1123.6 | $C_{53}H_{89}O_{22}$ | 1077.2 [M-H]$^-$,945.2[M-H-Ara(p)$^{5)}$]$^-$, 783.4 [M-H-Ara(p)-Glc]$^-$, 621.2 [M-H-Ara(p)-2Glc]$^-$, 459.2 [PPD aglycone-H]$^-$ |
| 12 | 8.10 | Rb3 | 1123.6 | $C_{53}H_{89}O_{22}$ | 1077.4 [M-H]$^-$,945.5 [M-H-Ara(p)$^{5)}$]$^-$, 783.2 [M-H-Ara(p)-Glc]$^-$, 621.4 [M-H-Ara(p)-2Glc]$^-$, 459.5 [PPD aglycone-H]$^-$ |
| 13 | 8.29 | Rt1 | 925.5 | $C_{47}H_{73}O_{18}$ | 763.2 [M-H-Glc]$^-$, 701.2 [M-H-(Glc-$CO_2$)-$H_2O$]$^-$, 613.1 [M-H-Glc-Ara-$H_2O$]$^-$, 587.2 [M-H-Ara-(Glc-$CO_2$)]$^-$, 569.3 [M-H-Ara-(Glc-$CO_2$)-$H_2O$]$^-$, 455.4 [OA aglycone-H]$^-$, |

| | | | | | |
|---|---|---|---|---|---|
| 14 | 9.86 | F1 | 683.4 | $C_{36}H_{61}O_9$ | 637.2 [M-H]$^-$, 475.4 [PPT aglycone-H]$^-$ |
| 15 | 10.80 | Chikusetsu Iva | 793.4 | $C_{42}H_{65}O_{14}$ | 631.4 [M-H-Glc]$^-$, 613.1 [M-H-Glc-$H_2O$]$^-$, 587.2 [M-H-Glc-$CO_2$]$^-$, 569.4 [M-H-Glc-$CO_2$-$H_2O$]$^-$, 455.4 [OA aglycone-H]$^-$, |
| 16 | 11.22 | Rd | 991.5 | $C_{48}H_{81}O_{18}$ | 945.5 [M-H]$^-$, 783.5 [M-H-Glc]$^-$, 765.1 [M-H-Glc-$H_2O$]$^-$, 621.4 [M-H-2Glc]$^-$, 459.4 [PPD aglycone-H]$^-$ |
| 17 | 14.99 | NG-Fe | 961.5 | $C_{47}H_{79}O_{17}$ | 915.4 [M-H]$^-$, 783.2 [M-H-Ara]$^-$, 621.2 [M-H-Ara-Glc]$^-$, 459.4 [PPD aglycone-H]$^-$ |
| 18 | 15.34 | Rd2 | 961.5 | $C_{47}H_{79}O_{17}$ | 915.2 [M-H]$^-$, 621.2 [M-H-Ara-Glc]$^-$, 459.4 [PPD aglycone-H]$^-$ |
| 19 | 15.82 | F4 | 811.5 | $C_{42}H_{69}O_{12}$ | 765.1[M-H]$^-$, 619.1 [M-H-Rha]$^-$, 457.1[PPD aglycone-2H] |
| 20 | 16.26 | F2 | 829.5 | $C_{42}H_{71}O_{13}$ | 783.2 [M-H]$^-$, 621.2 [M-H-Glc]$^-$, 603.2 [M-H-Glc-$H_2O$]$^-$, 459.4 [PPD aglycone-H]$^-$ |
| 21 | 16.91 | Rg3 | 829.5 | $C_{42}H_{71}O_{13}$ | 783.1 [M-H]$^-$, 621.4 [M-H-Glc]$^-$, 459.5 [PPD aglycone-H]$^-$, 375.6[PPD aglycone-H-C6H12]$^-$ |
| 22 | 19.02 | CK | 667.4 | $C_{32}H_{61}O_8$ | 621.2 [M-H]$^-$, 459.1 [PPD aglycone-H]$^-$, 375.1 [PPD aglycone-H-$C_6H_{12}$]$^-$ |

**Table S2**. Precision, stability, and recovery of 22 saponins in P. quinquefolium cultivated in Heilongjiang province.

| Peaks | Precisions RSD (%) | | Stability | Recovery | | | |
|---|---|---|---|---|---|---|---|
| | Intra-day (n=6) | Inter-day (n=6) | RSD, (n=5) | 80% | 100% | 120% | RSD(%) |
| **1** | 0.74 | 1.83 | 0.81 | 100.0 | 99.9 | 99.9 | 0.06 |
| **2** | 1.83 | 1.76 | 1.67 | 100.7 | 99.3 | 100.4 | 0.74 |
| **3** | 2.12 | 2.74 | 3.86 | 100.1 | 98.8 | 99.9 | 0.7 |
| **4** | 0.36 | 0.61 | 1.06 | 99.9 | 100.1 | 101.0 | 0.58 |
| **5** | 1 | 1 | 0.46 | 98.8 | 105.0 | 101.0 | 3.09 |
| **6** | 0.19 | 0.31 | 0.34 | 100.6 | 99.1 | 100.4 | 0.81 |
| **7** | 0.4 | 0.48 | 1.22 | 100.3 | 105.4 | 100.2 | 2.92 |
| **8** | 2.26 | 2.52 | 3.55 | 99.7 | 99.7 | 101.1 | 0.81 |
| **9** | 0.83 | 0.65 | 0.89 | 103.0 | 98.3 | 100.7 | 2.33 |
| **10** | 0.36 | 0.4 | 0.41 | 100.4 | 96.4 | 103.1 | 3.37 |
| **11** | 0.67 | 0.84 | 0.83 | 100.0 | 100.2 | 99.9 | 0.15 |
| **12** | 0.36 | 0.45 | 0.38 | 99.8 | 102.0 | 103.0 | 1.61 |
| **13** | 2.72 | 2.29 | 2.81 | 100.0 | 100.4 | 99.4 | 0.5 |
| **14** | 0.57 | 0.68 | 0.64 | 101.2 | 100.6 | 100.5 | 0.38 |
| **15** | 0.24 | 0.26 | 0.31 | 102.0 | 98.9 | 100.9 | 1.56 |
| **16** | 0.28 | 0.82 | 0.27 | 99.9 | 100.2 | 100.1 | 0.15 |
| **17** | 0.35 | 0.72 | 0.4 | 98.9 | 100.6 | 100.7 | 1.01 |
| **18** | 0.98 | 1.62 | 1.05 | 100.2 | 100.3 | 100.2 | 0.06 |
| **19** | 0.28 | 0.31 | 0.31 | 101.3 | 97.6 | 99.0 | 1.88 |
| **20** | 0.83 | 0.92 | 1.01 | 100.6 | 99.7 | 99.8 | 0.49 |
| **21** | 0.14 | 1.71 | 0.19 | 98.7 | 100.6 | 102.4 | 1.84 |
| **22** | 0.25 | 0.27 | 0.29 | 98.8 | 105.0 | 101.0 | 3.09 |

**Table S3.** Contents (μg/g) of 22 investigated compounds from different medicine parts and growth years in *P. quinquefolium* cultivated in Heilongjiang province.

| Analysts | 1 | 2 | 3 | 4 | 5 | 6 | 7 | 8 | 9 | 10 | 11 | 12 | 13 | 14 | 15 | 16 | 17 | 18 | 19 | 20 | 21 | 22 |
|---|---|---|---|---|---|---|---|---|---|---|---|---|---|---|---|---|---|---|---|---|---|---|
| **Leaves** | 5962 | 8186 | 21 | 6992 | 1773 | 21 | 87 | 1879 | 27 | 31 | 3134 | 5841 | 8 | 37 | 17 | 4046 | 709 | 1122 | 23 | 2421 | 0 | 16 |
| **Stems** | 2103 | 685 | 17 | 9678 | 2201 | 0 | 5 | 42 | 4 | 0 | 1215 | 3765 | 7 | 0 | 12 | 2755 | 20 | 889 | 8 | 99 | 0 | 0 |
| **Main roots** | 15184 | 1864 | 101 | 6529 | 34165 | 13 | 0 | 3457 | 5 | 14 | 2489 | 513 | 23 | 2 | 93 | 358 | 55 | 254 | 5 | 46 | 6 | 0 |
| **Hairy roots** | 33908 | 4427 | 111 | 8744 | 68628 | 64 | 12 | 2499 | 8 | 18 | 4722 | 3840 | 16 | 8 | 111 | 623 | 776 | 838 | 5 | 620 | 0 | 24 |
| **Rihizomes** | 23521 | 2695 | 56 | 5738 | 31260 | 35 | 16 | 676 | 4 | 0 | 1138 | 1878 | 46 | 4 | 684 | 3291 | 80 | 149 | 6 | 71 | 4 | 12 |
| **1yr** | 16189 | 5042 | 26 | 9189 | 14041 | 26 | 5 | 1567 | 5 | 41 | 1803 | 220 | 38 | 5 | 42 | 2548 | 16189 | 253 | 4 | 71 | 8 | 4 |
| **1yr** | 15874 | 4137 | 26 | 8130 | 15042 | 23 | 6 | 1608 | 5 | 33 | 1540 | 224 | 35 | 6 | 45 | 2261 | 15874 | 326 | 3 | 97 | 8 | 6 |
| **1yr** | 17127 | 5289 | 24 | 8249 | 13511 | 27 | 5 | 1482 | 5 | 33 | 1542 | 227 | 32 | 5 | 47 | 2314 | 17127 | 389 | 4 | 98 | 8 | 4 |
| **1yr** | 17594 | 5660 | 27 | 8822 | 15369 | 26 | 5 | 1630 | 5 | 38 | 1644 | 221 | 39 | 5 | 45 | 2500 | 17594 | 388 | 4 | 88 | 8 | 5 |
| **1yr** | 19039 | 4662 | 25 | 8519 | 13136 | 27 | 5 | 1723 | 5 | 40 | 1650 | 214 | 37 | 8 | 46 | 2381 | 19039 | 335 | 5 | 89 | 8 | 4 |
| **2yr** | 35807 | 1814 | 89 | 11714 | 42537 | 30 | 5 | 3679 | 14 | 27 | 2215 | 272 | 35 | 6 | 680 | 2619 | 39873 | 79 | 8 | 46 | 6 | 7 |
| **2yr** | 30986 | 1501 | 71 | 12101 | 31689 | 29 | 4 | 2492 | 13 | 13 | 2086 | 251 | 35 | 3 | 679 | 2643 | 36094 | 48 | 5 | 39 | 8 | 5 |
| **2yr** | 35287 | 1788 | 67 | 11601 | 43317 | 28 | 4 | 3220 | 15 | 13 | 2577 | 258 | 35 | 4 | 705 | 2779 | 37689 | 25 | 4 | 41 | 7 | 3 |
| **2yr** | 35574 | 1985 | 79 | 11201 | 44137 | 26 | 6 | 3576 | 14 | 26 | 2621 | 272 | 35 | 5 | 648 | 2926 | 32224 | 98 | 8 | 41 | 6 | 3 |
| **2yr** | 37608 | 1748 | 61 | 11154 | 36316 | 29 | 4 | 2474 | 13 | 15 | 1193 | 271 | 35 | 4 | 711 | 2891 | 39984 | 53 | 7 | 48 | 6 | 2 |
| **3yr** | 39873 | 4064 | 92 | 5720 | 68683 | 17 | 5 | 1707 | 8 | 7 | 1204 | 160 | 17 | 4 | 179 | 2980 | 35807 | 76 | 6 | 53 | 6 | 4 |
| **3yr** | 36094 | 2923 | 94 | 5325 | 60646 | 19 | 5 | 1619 | 7 | 9 | 852 | 143 | 24 | 4 | 132 | 3068 | 30986 | 89 | 6 | 55 | 6 | 5 |
| **3yr** | 37689 | 3484 | 91 | 6288 | 75171 | 18 | 4 | 1646 | 7 | 8 | 874 | 144 | 27 | 3 | 150 | 3068 | 35287 | 92 | 5 | 58 | 4 | 3 |
| **3yr** | 32224 | 5009 | 94 | 6801 | 80084 | 18 | 5 | 1744 | 8 | 8 | 851 | 141 | 32 | 3 | 176 | 3118 | 35574 | 103 | 5 | 57 | 5 | 4 |
| **3yr** | 39984 | 3497 | 91 | 5345 | 72696 | 18 | 4 | 1474 | 7 | 6 | 874 | 166 | 32 | 3 | 135 | 3172 | 37608 | 77 | 6 | 59 | 7 | 2 |
| **4yr** | 20080 | 3799 | 17 | 8655 | 77011 | 16 | 3 | 1388 | 8 | 16 | 686 | 95 | 63 | 3 | 173 | 2038 | 20080 | 22 | 4 | 39 | 8 | 4 |
| **4yr** | 17611 | 4011 | 21 | 7420 | 73456 | 15 | 3 | 1316 | 8 | 15 | 633 | 100 | 54 | 2 | 147 | 2000 | 17611 | 26 | 5 | 38 | 8 | 5 |
| **4yr** | 24158 | 4391 | 19 | 10134 | 82453 | 14 | 3 | 1459 | 8 | 15 | 623 | 116 | 64 | 2 | 174 | 2150 | 24158 | 23 | 5 | 41 | 7 | 6 |

| | | | | | | | | | | | | | | | | | | | | | |
|---|---|---|---|---|---|---|---|---|---|---|---|---|---|---|---|---|---|---|---|---|---|
| **4yr** | 25207 | 4140 | 28 | 9658 | 80577 | 14 | 3 | 1482 | 8 | 17 | 717 | 96 | 64 | 3 | 160 | 2206 | 25207 | 26 | 5 | 40 | 7 | 3 |
| **4yr** | 24082 | 3729 | 18 | 9505 | 80361 | 16 | 4 | 1439 | 9 | 15 | 758 | 91 | 56 | 2 | 157 | 2006 | 24082 | 25 | 4 | 40 | 7 | 5 |
